# Application of Solid-Supported Amines for Thermocatalytic Reactive CO_2_ Capture

**DOI:** 10.1021/acsomega.4c10049

**Published:** 2025-01-16

**Authors:** W. Wilson McNeary, Nathan C. Ellebracht, Melinda L. Jue, Mathew J. Rasmussen, James M. Crawford, Matthew M. Yung, Anh T. To, Simon H. Pang

**Affiliations:** † Catalytic Carbon Transformation and Scale-Up Center, National Renewable Energy Laboratory, Golden, Colorado 80401, United States; ‡ Materials Science Division, Lawrence Livermore National Laboratory, Livermore, California 94550, United States; § Department of Chemical & Biological Engineering, Montana State University, Bozeman, Montana 59717, United States

## Abstract

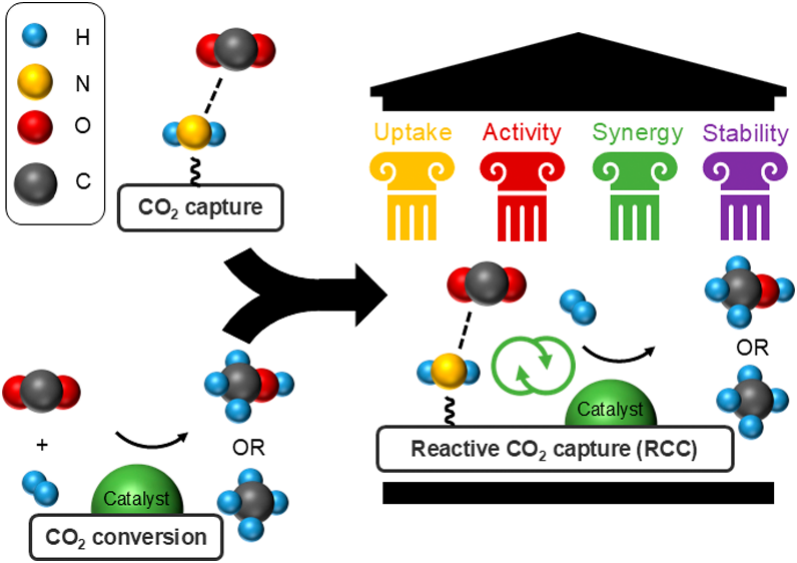

Reactive CO_2_ capture (RCC) is a promising strategy for process intensification of carbon capture and conversion for production of low-carbon fuels and chemicals. As state-of-the-art sorbent materials in point source and direct air capture systems, solid-supported amines are a natural choice to pair with supported CO_2_ hydrogenation catalysts (e.g., metallic nanoparticles) for developing high-capacity sorbent-catalyst materials for use in RCC. In this Perspective, we summarize the relevant literature combining solid-supported amines with metallic nanoparticles for thermocatalytic RCC and detail two of our own case studies using RCC to synthesize methane and methanol. Our observations suggest that the temperature mismatch between CO_2_ desorption and reaction, along with potential catalyst site poisoning by grafted aminosilanes, is a significant obstacle to realizing the potential of amine-based RCC materials in the decarbonization of chemical production. This stands in contrast to literature detailing successful RCC using liquid amines and solid catalysts, which may benefit from more favorable mass transfer dynamics, as well as early stage reports into RCC solid-phase amine-Pd materials, whose findings we were not able to replicate. More judicious reaction selection and synthetic design strategies to match materials with process conditions offer alternative pathways for future research.

## Introduction

Carbon dioxide (CO_2_) abatement and removal technologies will play an essential role in limiting global warming to <2 °C this century.^1^ Capture and subsequent storage of CO_2_ in underground reservoirs is a technologically mature but not yet broadly deployed approach that is being pioneered in locations with favorable geology for storage; however, the economic incentive for this approach relies on the coestablishment of a carbon market, which is still in its fledgling stages. By developing CO_2_ capture and conversion technologies, low-carbon and carbon-neutral fuels and chemicals can be synthesized and used within existing infrastructure to reduce near-term emissions in difficult-to-decarbonize sectors such as heavy transport, aviation, and industrial heating. Additionally, commodity chemicals synthesized from CO_2_ offer a fungible product to offset capture costs and have the added benefit of displacing identical fossil-derived chemicals. Currently, most proposed capture and conversion schemes involve discrete and separated steps in which the CO_2_ is captured by a sorbent or solvent, released through a regeneration process, compressed, potentially transported to another facility (as CO_2_ point sources may not be colocated with conversion facilities or the renewable energy resources required), and then converted into products via thermo- or electrocatalysis. Each step in the process (especially CO_2_ desorption for sorbent/solvent regeneration and CO_2_ compression) incurs a significant energy penalty, challenging the already difficult economics of valorizing a fully oxidized waste product. Transportation of the CO_2_ intermediate is also a nontrivial concern, as no significant pipeline infrastructure for CO_2_ exists today, and attempts to build such a network are the subject of regulatory hurdles, safety concerns, and public perception risks.^2^ Alternate methods of liquified CO_2_ transportation, such as truck, rail, and barge add significant expense and operational complexity to any carbon capture and utilization scheme and may still create public safety concerns.^3^ Process intensification could be achieved through reactive CO_2_ capture (RCC), in which CO_2_ is captured from a dilute gas source by a solvent or sorbent and subsequently converted into a desired product without dedicated separation, concentration/purification, and compression stages. This may offer significant energy and capital cost reductions of over 50% compared to separate capture and conversion by avoiding the above steps and using the catalytic conversion to simultaneously regenerate the solvent or sorbent.^4^ Conversion of the captured CO_2_ in a single unit operation also eliminates dedicated CO_2_ transport and storage requirements. Additionally, from a thermodynamic standpoint, RCC may access favorable intermediates in ways inaccessible to separate capture and conversion processes.^4^

Due to its ability to simultaneously address multiple drawbacks to conventional CO_2_ conversion, the development of sorbent-catalyst materials for RCC has become an area of increasing interest in recent years. One well-studied approach is the combination of alkaline oxides (e.g., Na_2_O, CaO) for CO_2_ adsorption with metals that are effective methanation catalysts (e.g., Ru, Ni) on a metal oxide carrier.^5,6^ The identity of the catalyst component can also be tuned to target carbon monoxide^7,8^ or methanol^9^ using Pt or Cu, respectively. While these materials can be highly selective, they often require temperatures >250 °C for RCC operation, similar to their standalone CO_2_ conversion catalyst counterparts, which increases the energy demand of the process and makes their use most appealing with midtemperature CO_2_ sources of 200 to 800 °C (e.g., power plant flue gas).^10^ The use of supported alkaline oxides in RCC contrasts with many state-of-the-art point source and direct air capture (DAC) systems (e.g., those being piloted by Climeworks),^11^ which instead utilize solid-supported amines due to their relatively high capacity and rapid adsorption/desorption kinetics at temperatures ≤100 °C. As such, amine-based approaches to RCC have the potential to expand the suite of available RCC materials and improve the flexibility of the process for lower temperature applications that are of increasing interest, including DAC.

**Figure 1:**
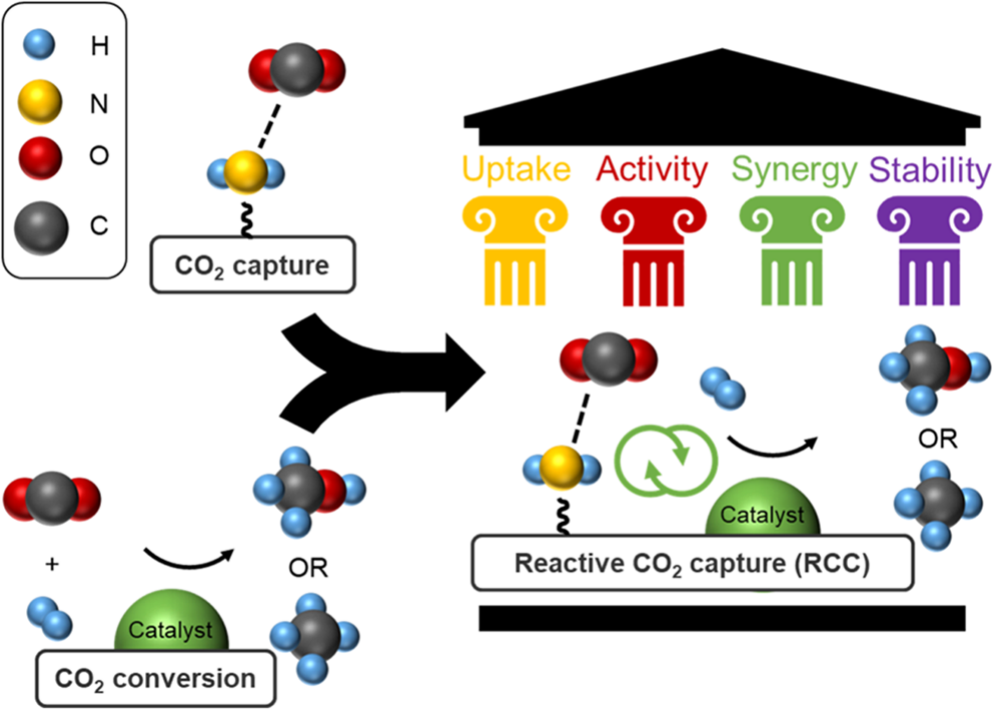
Effective materials for reactive CO_2_ capture will have high and rapid CO_2_ uptake, high catalytic activity and selectivity to the desired product, synergistic interactions between the capture and conversion sites, and long-term stability under all process conditions.

Utilizing the full CO_2_ capacity of a solid supported amine sorbent for RCC presents an opportunity to achieve dramatically higher product yield simply due to the greater CO_2_ uptake and more rapid adsorption kinetics possible with these sorbents at low temperatures compared to dispersed alkaline oxides^12^—though alkaline oxide sorbents can exhibit similar low-temperature properties through synergistic interactions with water.^13^ Additionally, the chemical properties of organic amines immobilized on a high-surface area support, such as organic linker length,^14^ amine degree of substitution,^15^ and cooperative interactions,^16^ offers a greater degree of tunability and freedom to potentially promote synergistic effects with a catalyst site. The temperature range under which the amines themselves are thermally stable under nonoxidizing environments (up to 250 °C)^17^ coincides with the temperature at which exothermic products like methane and methanol are thermodynamically preferred in catalytic CO_2_ hydrogenation.^18^ Most appealingly, different reaction pathways with lower activation energies may be accessible through the hydrogenation of amine-bound CO_2_ (e.g., carbamate), which is more electronically favorable for reduction compared to linear CO_2_ or alkaline carbonates.^19^ This could create opportunities to lower the reaction temperature during RCC and lessen energy costs associated with temperature swing, especially under DAC conditions. These potential advantages allow us to conceptualize the desired properties of an amine-based RCC material—high and rapid CO_2_ uptake at the basic amine site, a highly active and selective catalytic site, synergy between the proximate amine and catalytic sites, and stability of the material over many RCC cycles (Figure 1). To maximize uptake in RCC materials, inspiration can be drawn from the wealth of literature on optimization of amine-based CO_2_ sorbents.^20−22^ Similarly, strategies for increasing material stability can be developed from the solid amine sorbent field;^23^ however, additional considerations should be factored in for repeated thermal cycling to the higher temperatures required in RCC compared to conventional sorbent regeneration.^17,24,25^ Given the relative novelty of these systems, the remaining pillars of achieving high activity and synergy between the catalytic and basic amine sites are the least understood, though some proofs of concept are available in the literature.

A select number of promising reports have emerged in recent years that lend credence to aspects of utilizing amine-based RCC materials. Amine-containing phosphonic-acid monolayers have been used to enhance the performance of supported Pt and Pd catalysts in continuous CO_2_ hydrogenation by promoting stronger CO_2_ adsorption compared to unmodified catalysts,^26^ demonstrating the type of cooperative effects that may be utilized in an RCC configuration. Solid-supported amines have been demonstrated in tandem with homogeneous Ru catalysts in a solution of tetrahydrofuran for RCC to methanol (up to 34% yield).^27^ Additionally, liquid alkanolamines have been used as “relay molecules” for CO_2_ capture and hydrogenation to methane over Ru-MOF catalysts.^28^ Multiple reports have also demonstrated the use of an amine-based capture solvent (*N*-(2-ethoxyethyl)-3-morpholinopropan-1-amine; 2-EEMPA) to capture CO_2_, followed by condensed-phase hydrogenation to methane or methanol over heterogeneous catalysts (170 °C; *P*_H_2__ = 60 bar).^29,30^ This capture solvent approach is notable due to observation of a distinct reaction pathway through an *N*-formamide intermediate, which undergoes C–N cleavage to form the products, hinting at possible synergies between CO_2_-laden amines and supported metal nanoparticles. However, it is important to point out that in these condensed-phase approaches, the CO_2_ is being provided to the catalyst surface at a constant concentration through the flow of CO_2_-saturated 2-EEMPA, which creates favorable reactor dynamics akin to steady-state CO_2_ hydrogenation. The conversion component of RCC with solid-phase sorbent-catalysts is envisioned as a transient process, in which the bound CO_2_ is consumed over the course of the hydrogenation step and only replenished in the following capture step.

The first demonstrations of an entirely solid-phase approach have utilized Pd nanoparticles supported on silica that were grafted with aminopropyltriethoxysilane (APTES) or aminopropyltrimethoxysilane (APTMS).^31,32^ These materials were reported to be entirely selective to methanol and water under hydrogenation at 140 °C and atmospheric pressure. Up to 10% conversion per RCC cycle based on total CO_2_ adsorption capacity was achieved, indicating that much of the initially captured CO_2_ was desorbed unreacted. Diffuse reflectance infrared Fourier transform spectroscopy (DRIFTS) performed at various points during the RCC cycle indicated that CO_2_ was first adsorbed on the amine as a carbamate and then sequentially hydrogenated to an amide intermediate and finally methanol through hydrogen dissociation and transfer from the Pd sites.^31^ These are highly promising findings that necessitate replication and further study of the material properties.

This Perspective summarizes the nascent state of solid-supported amine materials for use in RCC and suggests focus areas for continued research. Here, we present two proof-of-concept case studies from our own work to build upon the aforementioned results and identify critical hurdles to the success of solid-supported amine-based RCC materials in the production of fuels and chemicals. While, in theory, these are highly promising RCC sorbents, we show that challenges around desorption/reaction temperature mismatch and catalytic site poisoning by steric effects, multilayer siloxane polymerization, and/or metal-N interactions limit the full potential of these materials. By no means is this work intended as a comprehensive study on the development of solid-supported amine-based RCC materials but serves as a timely account of results to help guide future research efforts.

## Methods

Detailed descriptions of the materials and methods used can be found in the Supporting Information. In brief, diamine-Ru materials for RCC to methane were synthesized by grafting *N*-(2-aminoethyl)-3-aminopropyltrimethoxysilane (“diaminosilane”) onto Ru-loaded catalysts. Amine-Pd materials for RCC to methanol were synthesized by grafting APTES onto Pd-loaded catalysts. Each class of materials underwent a variety of physicochemical characterization (elemental analysis, CO_2_ uptake, etc.) as well as reaction testing (steady-state CO_2_ hydrogenation, cyclic RCC). Specific testing conditions are provided alongside results in the Case Studies below.

## Case Studies

### Diamine-Ru Materials for RCC to Methane

In this first example, Ru-based RCC materials were explored for the conversion of CO_2_ to methane. A series of titania-supported diamine- and Ru-functionalized materials were prepared and probed for their CO_2_ adsorption and catalytic efficiency. The catalytic activity for methanation of these hybrid materials was directly compared to that of catalysts without the additional amine functionality. Titania microspheres (500 μm diameter, Johnson Matthey) were used as support materials. Catalysts prepared with 5 wt % nominal Ru loading (Table 1) were synthesized by strong electrostatic adsorption (SEA) Ru templating, and hybrid sorbent-catalyst materials were prepared by grafting diaminosilane to Ru-loaded catalysts using typical silane grafting procedures.^25^ Similar materials were additionally prepared with 1 wt % nominal Ru loading on amorphous silica pellets, to examine the impact of amine-to-Ru ratio and a nonreducible support, respectively; these are reported in the Supporting Information along with the full synthesis details.

**Table 1:** Grafted Aminosilane Loading and CO_2_ Capture Performance of Ru-Based Hybrid Sorbent-Catalyst Materials^a^

material	amine loading (μmol/g)^b^	Ru loading (wt %)^c^	12 h CO_2_ uptake (μmol/g)	amine efficiency (mol_CO_2__/mol_N_)	steady-state CO_2_ conversion (%)
diaminosilane/TiO_2_	990		170	0.17	3.8
5%Ru/TiO_2_		5	29		23
diaminosilane-5%Ru/TiO_2_	690	5	62	0.09	30

a CO_2_ uptake was performed at 30 °C in a dry 410 ppm of CO_2_/N_2_ environment. Steady-state conversion of CO_2_ performed at 200 °C with a H_2_:CO_2_ ratio of 6.67 and a CO_2_ partial pressure of 0.0057 bar.

b Amine loading determined by CHN elemental analysis. The associated grafted silane loading is half of the reported amine loading (two amine moieties per grafted silane molecule).

c Nominal Ru wt % for initial metal loading on support. Adjusted for additional mass from aminosilane grafting, the overall wt % decreases by ∼5%.

The presence of Ru partially reduced the extent of diaminosilane grafting compared to oxides without Ru, reducing the yield by 30–50% compared to diaminosilane-only materials (Tables 1 and S1). Based on typical oxide-grafted aminosilane densities,^16^ the silane functionalization resulted in at least monolayer oxide surface coverage with or without the presence of Ru. CO_2_ adsorption assessed via thermogravimetric analysis (TGA) indicated that the presence of Ru slightly reduced the amine efficiency, that is, the CO_2_ binding per grafted amine moiety. However, the reduced grafting yield meant sorbent-catalyst materials had lower overall CO_2_ uptake (Table 1). Specific CO_2_ capacities in these materials were primarily limited by their relatively low surface areas compared to mesoporous powders typically reported in the literature.^16^

An Altamira-300 reactor system was used to evaluate the performance of our materials as methanation catalysts under steady-state conversion. These experiments were performed at 200 °C with a H_2_/CO_2_ ratio of 6.67 and a CO_2_ partial pressure of 0.0057 bar, reported in Tables 1 and S1. Reaction conditions for the steady-state reaction are reported in Table S2. Conversion of CO_2_ to methane with the diaminosilane-5%Ru/TiO_2_ sorbent-catalyst was very similar to that of the metal-only catalysts, indicating that the addition of the aminosilane functionality did not hinder a Ru-catalyzed methanation pathway, especially with higher metal loadings. In comparison, the steady-state conversion of the diaminosilane-1%Ru/TiO_2_ was lower than that of the metal-only 1%Ru/TiO_2_, possibly indicating that highly dispersed Ru was blocked by silane networks or poisoned by strong association with amine moieties.

**Figure 2:**
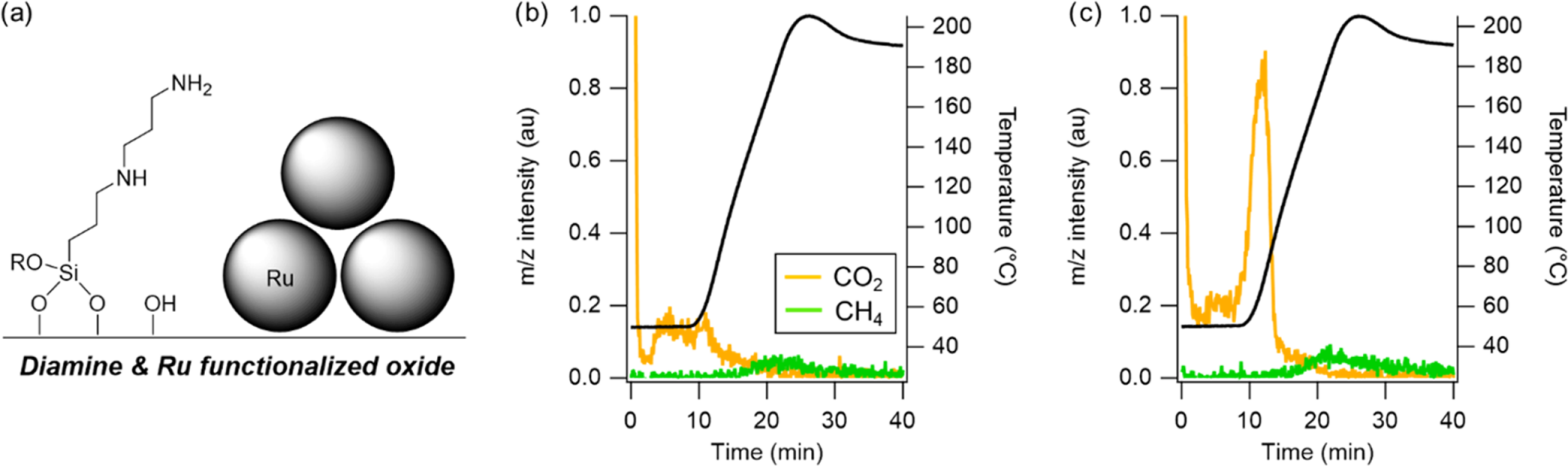
(a) Schematic of diamine-Ru RCC materials (Ru atoms depicted qualitatively to scale with grafted aminosilane). RCC product evolution under H_2_ flow at 10 bar_g_ with (b) diaminosilane-5%Ru/TiO_2_ and (c) diaminosilane-5%Ru/SiO_2_. More detail on the material from (c) can be found in the SI; RCC results included here for clarity on CO_2_ desorption during temperature ramp.

Performance evaluation of the materials under RCC conditions was conducted in a Micromeritics Effi microreactor. The RCC cycle consisted of initial reduction at 200 °C, CO_2_ loading at 50 °C for 30 min in 3 sccm CO_2_, 5 sccm Ar, and 92 sccm He, an inert gas purge, then heating to 200 °C at 10 °C/min in 5 sccm He, 5 sccm Ar, and 90 sccm H_2_ at 0, 5, or 10 bar_g_ total pressure. Mass spectrometer product evolution during the reaction portions of RCC cycles (10 bar_g_) are shown in Figure 2 for titania- and silica-supported diamine-Ru materials. In contrast to steady-state experiments, very low quantities of methane (<5% CO_2_ conversion) were produced for all materials under RCC cycle conditions. During the temperature ramp in H_2_, the majority of the CO_2_ was desorbed and lost via slip prior to detection of any methane; this is seen more clearly in the sharp CO_2_ desorption peak from the silica-based material (Figure 2c) at the beginning of heating. Additional RCC results with product evolution at 0 and 5 bar_g_ total pressure (Figure S1) show similar results, indicating that adjusting the pressure did not greatly affect CO_2_ slip. An analysis of the CO_2_ desorption from these materials is found in Figure S2. From those results and a plethora of literature studying amine-based CO_2_ sorbents, CO_2_ desorbs rapidly at temperatures well under 100 °C.^33^

The observed rapid CO_2_ desorption at temperatures well under 100 °C severely limits the utility of amine-based RCC materials for methanation. A small portion of the bound CO_2_ was converted to methane, but the majority was desorbed prior to reaching the light-off temperature. We initially hypothesized that amine-bound CO_2_ might be able to access lower activation energy pathways through cooperative interactions with the metal catalysts, but our results suggest that either these pathways do not exist or that the activation barrier remains too high, resulting in appreciable CO_2_ desorption before conversion. Without a method to kinetically stabilize the adsorbate (e.g., through the formation of a more thermally stable intermediate, extremely rapid heating, etc.), these amine-based materials, so far, are not promising for the reactive capture and conversion of CO_2_ to methane.

### Amine-Pd Materials for RCC to Methanol

As a second example, Pd-based RCC materials were used to convert CO_2_ into methanol. Based on the aforementioned reports of methanol production from Pd-amine RCC materials,^31,32^ similar materials were synthesized by grafting APTES onto SiO_2_-supported Pd catalysts. Details of this synthesis procedure are provided in the Supporting Information. The primary difference between these materials and those reported in Pazdera et al. is the addition of Pd to commercial SiO_2_ supports (Sipernat 22 and ACS Materials SBA-15) via strong electrostatic adsorption of tetraamine palladium(II) nitrate precursor rather than combined sol–gel synthesis of Pd/SiO_2_ using surfactant, organosilanes, and palladium acetylacetonate in water. Table 2 displays physicochemical properties of the synthesized materials expected to be relevant to their utility as RCC materials. In this case, in contrast to the diamine-Ru materials described above, steady-state CO_2_ hydrogenation data was not collected due to anticipated differences between the steady-state and RCC reaction mechanisms; however, CO chemisorption was performed on the materials to monitor Pd accessibility before and after APTES grafting.

**Table 2:** Grafted APTES Loading and CO_2_ Capture Performance of Pd-Based Hybrid Sorbent-Catalyst Materials^a^

material	amine loading^b^ (μmol/g)	Pd loading^c^ (wt %)	12 h CO_2_ uptake (μmol/g)	amine efficiency (mol_CO2_/mol_N_)	CO uptake^d^ (μmol/g)
5%Pd/SiO_2_		5%			42
3%Pd/SBA-15		3%			94
APTES-5%Pd/SiO_2_	1370	5%	470	0.34	8.4
APTES-3%Pd/SBA-15	3300	3%	1350	0.41	12

a CO_2_ uptake was performed at 30 °C in a 10% CO_2_/N_2_ environment. CO chemisorption performed at 50 °C under pulsed 10% CO/He.

b Amine loading determined by CHN elemental analysis. The associated grafted silane loading is equal to the reported amine loading (one amine group per grafted silane molecule).

c Nominal Pd wt % for initial metal loading on support.

d Samples pretreated under H_2_ flow at 200 °C for 2 h.

Relatively high amine loadings were achieved on the supported Pd catalysts, especially with SBA-15 as the support (likely due to its higher surface area and porosity over precipitated SiO_2_). Both APTES-grafted RCC materials were found to adsorb CO_2_, with APTES-3%Pd/SBA-15 possessing the highest CO_2_ uptake of 1346 μmol/g and an amine efficiency approaching the theoretical maximum of 0.5. Interestingly, CO chemisorption data indicates that the accessibility of Pd sites was significantly diminished on both 5%Pd/SiO_2_ and 3%Pd/SBA-15 after APTES grafting, with 80 and 87% of CO uptake lost, respectively.

**Figure 3:**
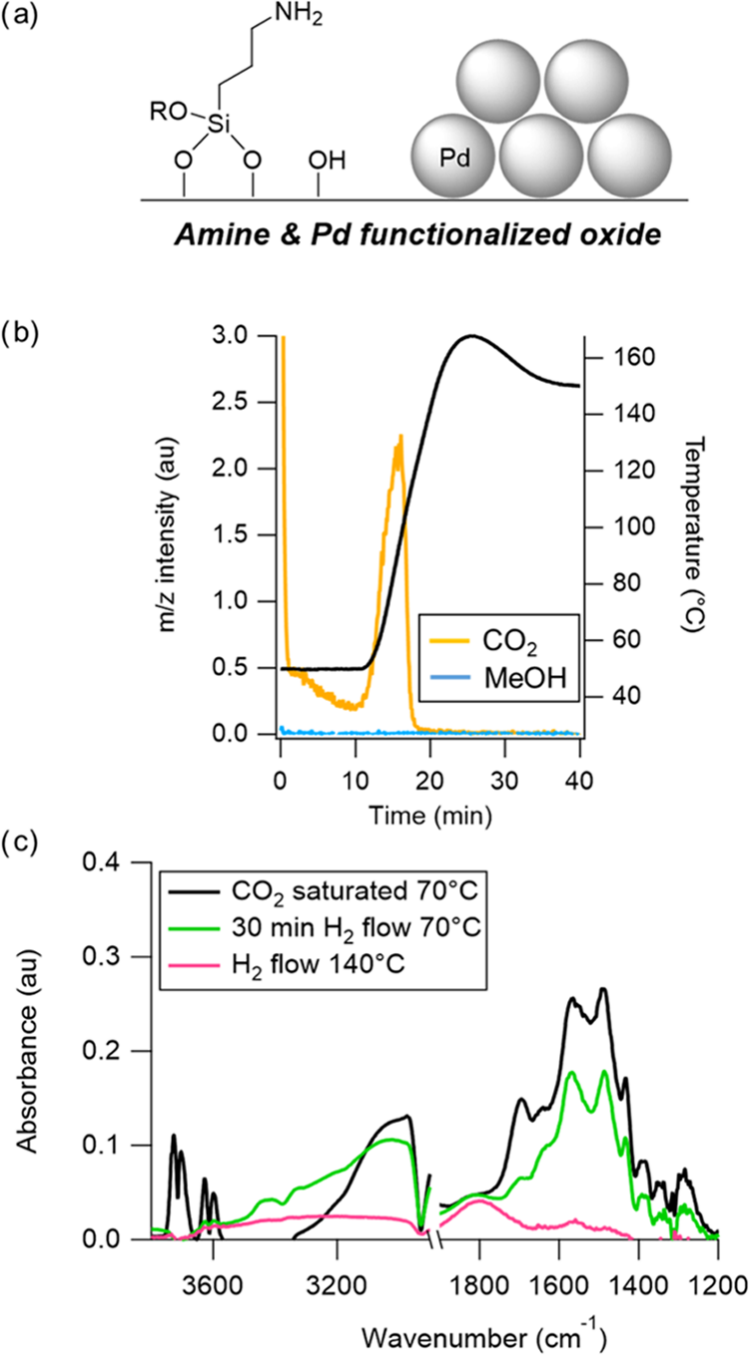
(a) Schematic of amine-Pd RCC materials (Pd atoms depicted qualitatively to scale with grafted aminosilane); (b) product evolution on APTES-3%Pd/SBA-15 under atmospheric H_2_ flow following CO_2_ adsorption; (c) DRIFTS spectra on APTES-5%Pd/SiO_2_ after CO_2_ saturation at 70 °C, following 30 min H_2_ flow at 70 °C, and after increasing the temperature to 140 °C under H_2_ flow (5 °C/min heating rate).

Given its high CO_2_ uptake, the APTES-3%Pd/SBA-15 sample was tested in an RCC-type cycle (Figure 3). First, the sample was pretreated under H_2_ flow at 190 °C. Then, the sample was cooled to 50 °C and exposed to 10% CO_2_/He at 100 sccm for 1 h. Flow was then switched to N_2_, and the reactor was purged for 10 min. Following this, the flow was switched to 100 sccm H_2_, and the temperature was increased to 150 °C at 10 °C/min. In this case, this step was conducted at atmospheric pressure. A selection of mass spectrometry data for product evolution on this catalyst during the temperature-programmed hydrogenation is shown in Figure 3b. The sole species evolved was CO_2_ at 80 °C and none of the desired methanol was produced at any temperatures. Similar behavior was observed in other RCC experiments both with APTES-3%Pd/SBA-15 at ambient and intermediate pressures and the APTES-5%Pd/SiO_2_ (not reported here). These results stand in contrast to the observations in Pazdera et al.,^31,32^ in which an initial loss of CO_2_ during temperature-programmed hydrogenation was followed by production of methanol and H_2_O.

To further investigate the lack of activity on our amine-Pd RCC materials, DRIFTS spectra were collected on the APTES-5%Pd/SiO_2_ during a similar RCC-type experiment (Figure 3c). The conditions of these DRIFTS experiments were modeled after those conducted in Pazdera et al.^31^ Carbamate peaks were present upon CO_2_ saturation (1800–1400 cm^–1^), but those peaks were nearly eliminated during an inert purge due to CO_2_ desorption, and no hydrogenation of the remaining carbamates to amide intermediates (expected as a broad peak between 3700–2930 cm^–1^)^31^ was observed in the spectra collected under H_2_ flow, even at elevated temperatures.

Taken together, these results suggest that while our amine-Pd RCC materials readily capture CO_2_ as surface carbamates, the carbamates are not catalytically converted in any appreciable way by the Pd nanoparticles on the surface. One reason for this may be the rapid decomposition of the carbamates and release of CO_2_ at relatively low temperatures (80–100 °C) before sufficient temperatures are achieved for the Pd to activate any surface reactions. However, in the most relevant prior reports,^31,32^ conversion of carbamates to amides was reported even at 70 °C under H_2_ flow. Another factor may be the lack of access to the Pd surface post-APTES grafting, as evidenced by our chemisorption results in Table 2. While Pazdera et al. did not examine Pd accessibility in their materials, it stands to reason that multilayer siloxane networks, even if initiated on the silanol groups of the support, might sterically impede the Pd sites, resulting in a material with high CO_2_ uptake but little catalytic ability.

## Outlook

**Figure 4:**
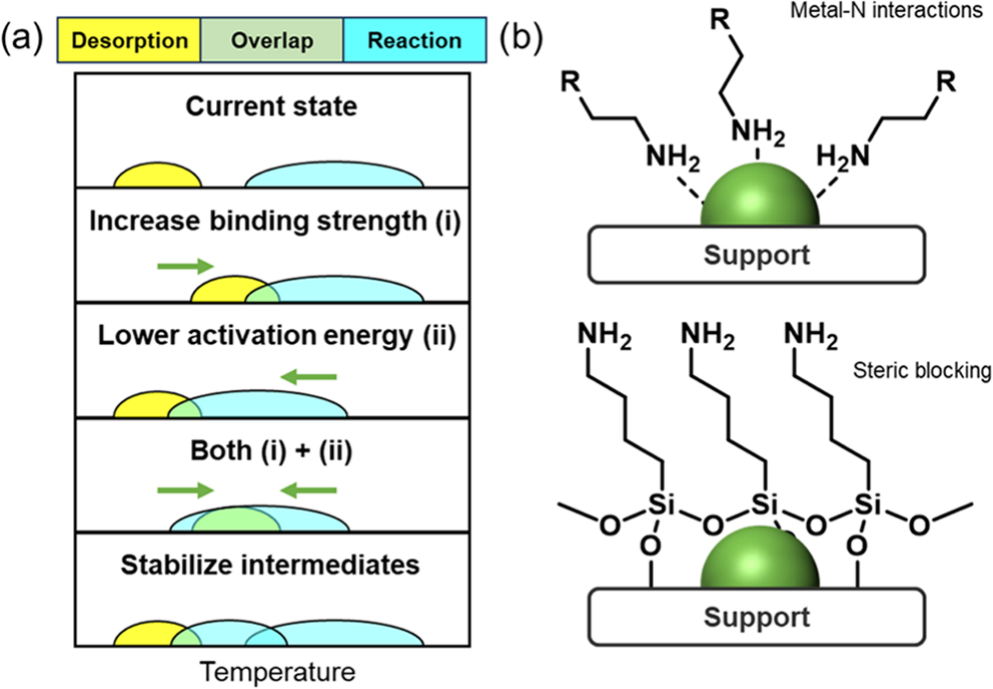
Though RCC holds promise for process intensification, there are several potential explanations why an amine-based RCC material may have poor performance: (a) mismatch between CO_2_ desorption temperature and reaction temperatures; (b) blocking of catalytic sites by grafted siloxane networks.

While solid-supported amines have great promise as energy efficient sorbent materials in both point source capture and DAC applications, our results show that it can be challenging to leverage their high affinity for CO_2_ when combined with a catalytic component in an RCC material. Our work suggests two potential reasons for this, the first being a mismatch between the thermal stability of surface-bound CO_2_ (e.g., carbamates) with the temperatures required for catalytic CO_2_ conversion (Figure 4a). In other words, we did not observe the hypothesized synergy between the capture and conversion capabilities of the material, nor was there sufficient adsorbate or intermediate stability to allow for efficient RCC. In both diamine-Ru/TiO_2_ and APTES-Pd/SiO_2_ materials presaturated with CO_2_, we observed significant CO_2_ desorption at <100 °C during temperature-programmed hydrogenation, indicating that most or all of the CO_2_ adsorbed on these samples was released without being converted to products. In this way, the facile regeneration properties of solid-amine sorbents in conventional CO_2_ capture schemes are detrimental to their potential as RCC materials. For amine-based sorbent-catalysts to be effective in RCC, it is essential that the CO_2_ be bound in a state that is thermally stable enough to reach catalytically relevant temperatures. We recommend further research into the design of alternative organic superbase moieties that may exhibit *stronger* CO_2_ binding energies to enable greater stability of bound CO_2_ intermediates at higher temperatures while retaining the chemical tunability of the organic component. Superbases such as amidine and guanidine can form stable carbamates and may also be molecularly tuned to alter CO_2_ release temperature.^34,35^ While traditionally explored as capture solvents,^36,37^ these functional groups can in some cases also be immobilized on solid supports,^38^ which suggests potential utility in the design of new, more effective sorbent-catalysts for RCC. Efforts in this area should be directed toward tethering the molecules to a range of catalyst support materials and understanding formation of intermediates under hydrogenation conditions, as these may be different from what has been reported in other amine-based RCC systems. Alternatively, the temperature mismatch could be bridged by accessing catalytic pathways with lower activation barriers by initially converting surface carbamates into more stable intermediates that can then undergo further hydrogenation to products as the temperature is increased. Such a phenomenon was suggested by Pazdera et al.^31,32^ but was unable to be replicated in our work, perhaps due to extreme sensitivity to the placement of grafted amines and catalytic nanoparticles across the surface. Process-based solutions that alter the activity may also be of use—if the RCC materials were designed to be compatible with near-instantaneous heating strategies such as photothermal, microwave, Joule, or magnetic induction heating, the rate of temperature increase between adsorption and reaction could be rapidly accelerated, thereby minimizing time spent at “intermediate” temperatures that are hot enough for desorption but not yet sufficient for activation of the bound CO_2_. An example of this rapid-thermal cycling RCC with Joule heating is being pioneered by Susteon with alkaline-based sorbent-catalysts and could be adapted for amine-based materials if the appropriate substrate is were chosen (i.e., one that is responsive to the heating method of interest).^39^ These alternative heating methods may also unlock lower-activation energy kinetic pathways for conversion by influencing electron arrangement at the catalyst sites, as has been recently observed for CO oxidation and the reverse water–gas shift reaction when powered by induction heating.^40^

A second contributing factor to the lack of product formation with amine RCC materials could be from a deleterious effect between the adsorption and catalytic functionalities in the form of unintentional catalyst site blocking resulting from the grafting of aminosilane molecules onto a heterogeneous catalyst surface (Figure 4b). In our case studies above, we chose to add catalytic metal first and graft aminosilanes second to avoid destruction of the amines during high-temperature calcination of metal precursors, a standard postprocessing step for metal nanoparticle catalyst synthesis. Even though aminosilanes would be expected to preferentially graft at hydroxyl sites on the catalyst support, it is possible that the catalyst nanoparticles could be sterically blocked by nearby grafted amine molecules, especially at higher amine loadings. It is also possible that metal-N interactions could block the catalyst sites, thereby also inactivating those amines for CO_2_ capture. The loss of CO uptake observed on our APTES-Pd/SiO_2_ materials after grafting suggests such phenomena may be relevant. Blocking of the catalyst sites by the grafted amines would be expected to impede the ability of the catalyst sites to dissociate H_2_ and impede any cooperative carbamate conversion mechanism between the amine and catalyst sites. Creative synthesis approaches are needed to ensure catalyst site accessibility in amine RCC materials. A promising route may be to simply reverse the synthesis order and add amines to the substrate first and catalytic nanoparticles last. Care should be taken to avoid synthesis methods that require thermal treatments, such as high-temperature calcination, to remove stabilizing ligands or precursor residues, as these will likely also degrade the grafted amines. We recommend investigation into surfactant-free nanoparticle addition approaches,^41,42^ which, although they typically do not afford the same control of nanoparticle size as more controlled colloidal synthesis methods, do not require postsynthesis thermal treatment. We also recommend research into surface functionalization strategies to promote selective grafting of the amines on the support while protecting the catalyst nanoparticles from deposition. Masking strategies using self-assembled monolayers applied only to the catalyst particles may be of use here, but, again, should be utilized only if the masks can be removed nonthermally as to not damage the grafted amines. It is also possible that if amine coverage of the catalyst sites could be precisely controlled (i.e., to a greater degree than in conventional solution-phase grafting), metal-N interactions could be harnessed to provide beneficial activity enhancements via changes to intermediate stability—an effect that has been observed in electrochemical CO_2_ reduction.^43^

As this Perspective has emphasized through two case studies, simple combinations of state-of-the-art adsorbent and catalyst materials may not result in effective materials for an intensified and integrated reactive capture process due to incompatibility between the components and with the process. Though amines are excellent for capturing CO_2_ from dilute sources, their low-temperature adsorption reversibility, an asset when considering a standalone capture process, makes them potentially incompatible with high-temperature catalytic conditions, absent clever reaction engineering. Judicious selection of reactions that can be operated at or near CO_2_ capture temperatures may result in higher CO_2_ conversion. Creative materials design and synthesis strategies may be used to more precisely position capture and catalytic functional groups on the support material and enable synergistic interactions, which could lower reaction energy barriers while not poisoning or blocking catalytic sites. Incorporating organic functional elements into a hybrid material for thermocatalytic RCC may still be feasible and merits further research, but this approach will require careful choices of material, reaction, and process.

## Supporting Information Available

Methods and materials for synthesis of diamine-Ru and amine-Pd sorbent-catalysts, and additional characterization, RCC, and temperature-programmed desorption results for the diamine-Ru materials is also included (PDF)
