# Supplementary material for: Application of Solid-Supported Amines for Thermocatalytic Reactive CO2 Capture
Source: ACS Omega. 2025 Jan 16;10(3):2364–71. doi: 10.1021/acsomega.4c10049 (PMC11780555; doi:10.1021/acsomega.4c10049)
Supplement: Supplementary file 1 — ao4c10049_si_001.pdf [file ao4c10049_si_001.pdf]

Supporting Information

Application of solid-supported amines for thermocatalytic reactive CO_2 capture

W. Wilson McNeary[*1], Nathan C. Ellebracht[2], Melinda L. Jue[2], Mathew J. Rasmussen[1], James M. Crawford[3], Matthew M. Yung[1], Anh T. To[1], Simon H. Pang*[*2]*

[1]Catalytic Carbon Transformation and Scale-Up Center, National Renewable Energy Laboratory, CO 80401, USA

[2]Materials Science Division, Lawrence Livermore National Laboratory, Livermore, CA 94550, USA

[3]Department of Chemical & Biological Engineering, Montana State University, Bozeman, MT 59717, USA

SUPPLEMENTAL METHODS AND RESULTS

Synthesis of materials for RCC of CO_2 to methane

Strong electrostatic adsorption (SEA) was employed for Ru templating on the oxide supports. In SEA, an alkaline solution imparts a negative surface charge to the TiO_2 or SiO_2 surface such that the Ru precursor is electrostatically attracted to the surface. DI water (>14 MΩ·cm) was adjusted to pH = 9 using aqueous ammonia (56.6 wt%, Sigma-Aldrich, ACS reagent). TiO_2 or SiO_2 was added to this alkaline water and a ruthenium precursor (hexaamineruthenium(II) chloride, Sigma Aldrich, 99.9% trace metal basis) was dissolved in a separate beaker of pH 9 DI water. Both solutions were allowed to equilibrate for 30 minutes before mixing. The Ru solution was added gradually to the TiO_2 or SiO_2 suspension through a burette under constant mixing. The combined solution was placed on a shaker plate at 150 rpm for 24 hours before removing the TiO_2 spheres or SiO_2 pellets and drying at 110 ˚C overnight. After drying, the samples were washed using a 0.1 M NH_4OH solution to remove chlorine impurities. Each sample was exposed to 500 mL/g of wash solution and redried under the same conditions. Finally, the catalysts were calcined in air at 300 ˚C for 3 h in a muffle furnace with a temperature increase rate of 10 ˚C/min.

For hybrid sorbent-catalyst materials, the Ru was templated onto the TiO_2 or SiO_2 before grafting of the diamine to avoid undesired amine degradation during the calcination step. The 1 and 5 wt% Ru-loaded catalysts, described above, as well as bare TiO_2 or SiO_2, were used as the starting materials. Prior to grafting, materials were dried overnight in a vacuum oven at 100 ˚C in Erlenmeyer flasks. Flasks were sealed under Ar and transferred to a shaker plate (100 rpm). 25 mL toluene per g support was added via syringe. After at least 30 min of agitation, 0.2 g DI H_2O per g

support was added via syringe and allowed to disperse and equilibrate for an hour before 3 mmol of *N*-(2-aminoethyl)-3-aminoproplytrimethoxysilane ("diaminosilane") per g support, dispersed in 1–2 mL of toluene, was introduced dropwise via syringe. The mixture was shaken (100 rpm) at room temperature (~22 °C) overnight. To recover, the solution was decanted from the solids, which were then rinsed with 100 mL per g each of methanol, toluene, hexane, and methanol again in a vacuum filter before drying at 80 °C in a vacuum oven.

Detailed results from diaminosilane grafting and CO_2 adsorption

The yields from diaminosilane grafting to Ru-decorated supports were evaluated via CHN elemental analysis (**Table 1** and **Table S1**); grafting using typical methods as described was successful in producing materials with high amine loading. SiO_2 supports were investigated to probe the effects of amine loading on RCC performance; the higher surface area SiO_2 supports allow for greater amine loading, with ~160 m^2/g and ~65 m^2/g for the SiO_2 and TiO_2 supports, respectively. However, the presence of Ru partially hindered the extent of diaminosilane grafting, reducing the yield by 30–50%. The silane yields on Ru-loaded catalysts represent average spatial grafting densities of 2.2–3.2 silane molecules per nm^2 support and were similar for both TiO_2 and SiO_2 support types, which reflects that the grafting yield is largely determined by the support surface area and grafting conditions. This density is greater than monolayer coverage based on other reports of oxide-grafted aminosilanes.[1,2] The presence of dispersed nanoparticles of Ru on the support could partially block the accessible surface for grafting, but silanes may also oligomerize by condensing to other surface-grafted silanes.

Table S1. Additional results for grafted aminosilane loading and CO_2 capture performance of Ru-based hybrid sorbent-catalyst materials.

Material	Amine loading (μmol/g)	Ru loading (wt%)	12h CO_2 uptake (μmol/g)	Amine efficiency (mol_{CO2}/mol_N)	Steady-state CO_2 conversion (%)
Diaminosilane/TiO_2	990[a]	--	170	0.17	3.8
1%Ru/TiO_2	--	1	58	--	31.5
Diaminosilane-1%Ru/TiO_2	490[a]	1	85	0.17	13.1
5%Ru/TiO_2	--	5	29	--	22.7
Diaminosilane-5%Ru/TiO_2	690[a]	5	62	0.09	29.9
Diaminosilane/SiO_2	2400[b]	--	510	0.21	n.d.
1%Ru/SiO_2	--	1	0	--	n.d.
Diaminosilane-1%Ru/SiO_2	1600[b]	1	340	0.21	n.d.
5%Ru/SiO_2	--	5	0	--	n.d.
Diaminosilane-5%Ru/SiO_2	1500[b]	5	320	0.21	n.d.

[a]Amine loading determined by CHN elemental analysis. The associated grafted silane loading is half of the reported amine loading (two amine moieties per grafted silane molecule).

[b]Silane grafting loading calculated from organic burnout mass loss in TGA during a 10 ˚C /min ramp to 900 ˚C in air. Amine loading as reported is twice the measured silane loading. Where elemental analysis and TGA burnout data were both available, the measured silane loadings were typically within 15%. Relative error in measuring silane loading tends to decrease with increased loading.

n.d. = not determined

The CO_2 adsorption by these materials under simulated air capture conditions was assessed via thermogravimetric analysis (TGA). Materials were degassed (of CO_2 and H_2O) at 100 ˚C in N_2 for 1 h prior to adsorption of 410 ppm of CO_2 in N_2 at 30 ˚C for 12 h. The pseudo-equilibrium CO_2 capacity and amine efficiency of each material are reported in **Table 1** and **Table S1**. The amine efficiency is a measure of the effectiveness of the grafted aminosilanes in binding CO_2. The amine efficiencies of most of the materials, with and without Ru, are indicative of typical grafted aminosilane CO_2 capture from air, which is often in the range of ~0.15–0.2.[1,3] Despite the reduced grafting yield, the 1% Ru hybrid sorbent did not exhibit a decrease in amine efficiency, indicating that the presence of Ru does not necessarily hinder the adsorption of CO_2. With higher Ru loading, the amine efficiency of CO_2 binding was roughly halved. This could be a result of strong interaction between grafted amine moieties and Ru nanoparticles. From these results, we demonstrated that TiO_2 is an effective, albeit low surface area, support for CO_2-binding aminosilanes, materials with both Ru and aminosilane functionalities may be readily synthesized on TiO_2 and SiO_2, and that the presence of Ru does not significantly hinder the binding of CO_2 from air.

Additional RCC of CO_2 to methane results at varying total pressure

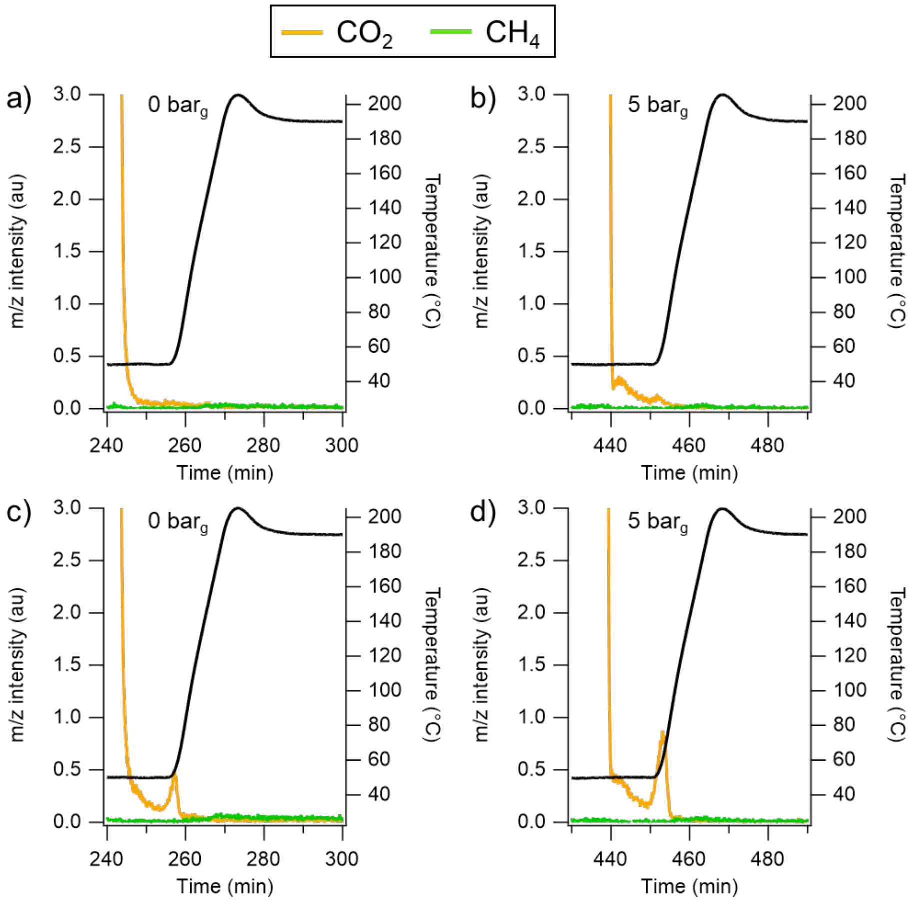

Figure S1. RCC product evolution under H_2 flow at 0 and 5 bar_g with a,b) diaminosilane-5%Ru/TiO_2 and c,d) diaminosilane-5%Ru/SiO_2.

CO_2 adsorption and temperature programmed desorption from sorbent-catalyst materials

In this reactor system, the hybrid sorbent-catalysts were first evaluated for their CO_2 uptake (**Table 1** and **Table S1**). After drying and reduction, a steady flow of 2% CO_2 (a 50-fold increase in p_{CO2} compared to air capture conditions) was passed over the material for 30 mins at 30 ˚C. The samples were purged with He for 30 mins and heated to 200 ˚C at 10 ˚C/min (**Figure S2**). The only desorbed products measured were CO_2 and water; no decomposition products were observed. Bare TiO_2 adsorbs a small amount of CO_2 at 30 ˚C, with the addition of Ru increasing the CO_2 adsorption

slightly (<10 μmol/g) due to strong interactions between CO_2 and Ru(0).[4] As in the CO_2 adsorption experiments, the introduction of diamine grafting significantly increased the CO_2 capacities, with the bare support showing an order of magnitude increase and a doubling of the capacity for the Ru-loaded materials. Competition between the Ru and diamine for grafting sites may contribute to the overall lowered CO_2 capacity relative to the Ru-free materials.

While heating in an inert environment (He), the bare TiO_2, diaminosilane-TiO_2, 1%Ru/TiO_2, and 5%Ru/TiO_2 materials all exhibit a single CO_2 desorption peak centered around ~50 ˚C that is fully desorbed by 100 ˚C. Interestingly, the diaminosilane-1%Ru/TiO_2 and diaminosilane-5%Ru/TiO_2 samples show a second CO_2 desorption peak centered around ~200 ˚C, suggesting that the presence of Ru promoted formation of a more strongly bound form of CO_2. For the diaminosilane-5%Ru/TiO_2 sample (**Figure S1f**), the second high temperature desorption peak accounted for nearly 40% of the total released CO_2. The reactor operating conditions for the steady-state methanation are reported in **Table S2.**

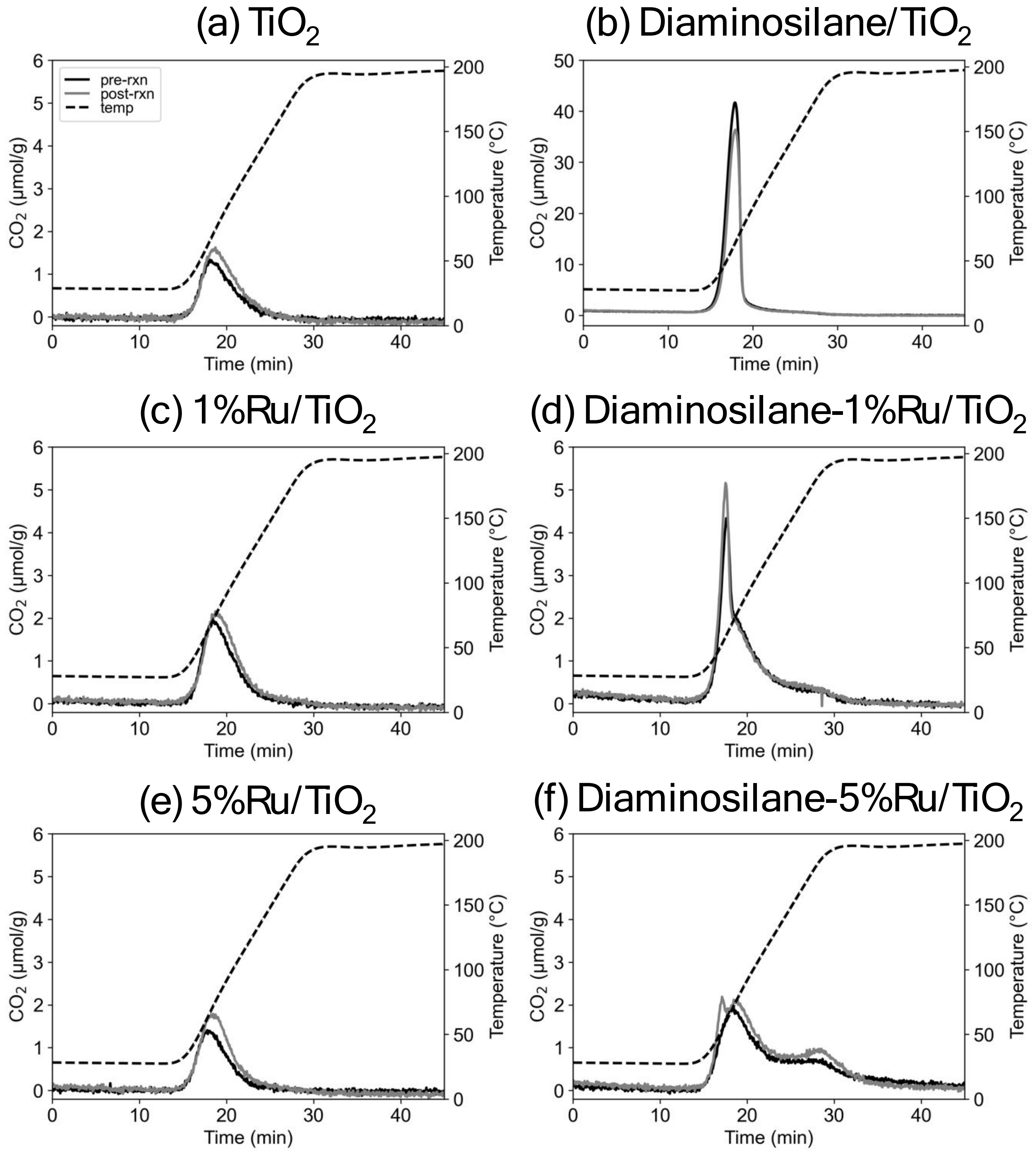

Figure S2. Temperature programmed desorption of CO_2 from a) TiO_2, b) diaminosilane-TiO_2, c) 1%Ru/TiO_2, d) diaminosilane-1%Ru/TiO_2, e) 5%Ru/TiO_2, f) diaminosilane-5%Ru/TiO_2 pre-reaction (black) or post-reaction (grey). The temperature profile is shown in the dashed line. Note

that subpanel b) is presented with a larger scale on the left vertical axis. Pre-reaction TPD data are from Steps 1–3 of the test series described in **Table S2**. Post-reaction TPD data are from steps 16–18 of Table S2, after the material has been reduced, run in a temperature ramp reactive capture process (Steps 5–7), run in steady state methanation conditions up to 200 ˚C (Steps 8–14), and then re-reduced (Step 15).

Table S2. Steady-state methanation conditions for Ru-diamine hybrid sorbent-catalyst materials.

Step	Description	Gas flow
1	Load with CO_2 for 30 min	10 sccm 10% CO_2/He + 40 sccm He
2	Purge with inert gas for 30 min	50 sccm He
3	Heat to 200 ˚C at 10 ˚C/min (CO_2 TPD)	50 sccm He
4	Reduce at 200 ˚C for 30 min	50 sccm 4% H_2/Ar
5	Cool to 30 ˚C and load with CO_2 for 30 min	10 sccm 10% CO_2/He + 40 sccm He
6	Purge with inert gas for 30 min	50 sccm He
7	Heat to 200 ˚C at 10 ˚C/min (CO_2 TPD)	50 sccm He
8	Reduce at 200 ˚C for 30 min	50 sccm 4% H_2/Ar
9	Cool to 30 ˚C and begin reactant flow	3 sccm 10% CO_2/He + 50 sccm 4% H_2/Ar
10	Stabilize MS signal in reactant flow, hold for 120 min	3 sccm 10% CO_2/He + 50 sccm 4% H_2/Ar
11	Heat to 125 ˚C under reactant flow, hold for 120 min	3 sccm 10% CO_2/He + 50 sccm 4% H_2/Ar
12	Heat to 150 ˚C under reactant flow, hold for 120 min	3 sccm 10% CO_2/He + 50 sccm 4% H_2/Ar
13	Heat to 175 ˚C under reactant flow, hold for 120 min	3 sccm 10% CO_2/He + 50 sccm 4% H_2/Ar
14	Heat to 200 ˚C under reactant flow, hold for 120 min	3 sccm 10% CO_2/He + 50 sccm 4% H_2/Ar
15	Reduce at 200 ˚C for 30 min	50 sccm 4% H_2/Ar

16	Cool to 30 ˚C and load with CO_2 for 30 min	10 sccm 10% CO_2/He + 40 sccm He
17	Purge with inert gas for 30 min	50 sccm He
18	Heat to 200 ˚C at 10 ˚C/min (CO_2 TPD)	50 sccm He

Synthesis of materials for RCC of CO_2 to methanol

Both a precipitated SiO_2 (Sipernat 22) and SBA-15 (ACS Materials) were used as supports, and Pd was added via strong electrostatic adsorption with tetraaminepalladium (II) nitrate as a precursor.[5] After Pd addition, the catalysts were calcined for 4 hours at 400 ˚C. For APTES grafting, the calcined material was dried at 110 ˚C overnight, then dispersed in 50 mL of toluene and heated to 80 ˚C. Once the temperature was reached, 0.2 mL of water was added and the solution was stirred for 1 h. At this point, 2 mL of APTES was added, and the mixture was further stirred for 16 h. The resultant material was filtered and washed with toluene and methanol in that order and finally dried at 110 ˚C for 4 h.

REFERENCES

(1) Yoo, C.-J.; Lee, L.-C.; Jones, C. W. Probing Intramolecular versus Intermolecular CO2 Adsorption on Amine-Grafted SBA-15. *Langmuir* **2015**, *31* (49), 13350–13360. https://doi.org/10.1021/acs.langmuir.5b03657.

(2) Didas, S. A.; Sakwa-Novak, M. A.; Foo, G. S.; Sievers, C.; Jones, C. W. Effect of Amine Surface Coverage on the Co-Adsorption of CO2 and Water: Spectral Deconvolution of Adsorbed Species. *J. Phys. Chem. Lett.* **2014**, *5* (23), 4194–4200. https://doi.org/10.1021/jz502032c.

(3) Didas, S. A.; Kulkarni, A. R.; Sholl, D. S.; Jones, C. W. Role of Amine Structure on Carbon Dioxide Adsorption from Ultradilute Gas Streams Such as Ambient Air. *ChemSusChem* **2012**, *5* (10), 2058–2064. https://doi.org/10.1002/cssc.201200196.

(4) Zağli, E.; Falconer, J. L. Carbon Dioxide Adsorption and Methanation on Ruthenium. *Journal of Catalysis* **1981**, *69* (1), 1–8. https://doi.org/10.1016/0021-9517(81)90122-6.

(5) Miller, J. T.; Schreier, M.; Kropf, A. J.; Regalbuto, J. R. A Fundamental Study of Platinum Tetraammine Impregnation of Silica: 2. The Effect of Method of Preparation, Loading, and Calcination Temperature on (Reduced) Particle Size. *Journal of Catalysis* **2004**, *225* (1), 203–212. https://doi.org/10.1016/j.jcat.2004.04.007.
